# New World Cactaceae Plants Harbor Diverse Geminiviruses

**DOI:** 10.3390/v13040694

**Published:** 2021-04-16

**Authors:** Rafaela S. Fontenele, Andrew M. Salywon, Lucas C. Majure, Ilaria N. Cobb, Amulya Bhaskara, Jesús A. Avalos-Calleros, Gerardo R. Argüello-Astorga, Kara Schmidlin, Anthony Khalifeh, Kendal Smith, Joshua Schreck, Michael C. Lund, Matias Köhler, Martin F. Wojciechowski, Wendy C. Hodgson, Raul Puente-Martinez, Koenraad Van Doorslaer, Safaa Kumari, Kehinde A. Oyeniran, Christian Vernière, Denis Filloux, Philippe Roumagnac, Pierre Lefeuvre, Simone G. Ribeiro, Simona P. Kraberger, Darren P. Martin, Arvind Varsani

**Affiliations:** 1 The Biodesign Center for Fundamental and Applied Microbiomics, Arizona State University, Tempe, AZ 85287, USA; rafasfontenele@asu.edu (R.S.F.); ilaria.cobb@gmail.com (I.N.C.); amulyabhaskara@gmail.com (A.B.); kara.schmidlin@asu.edu (K.S.); akhalif5@asu.edu (A.K.); krsmit39@asu.edu (K.S.); jrschrec@asu.edu (J.S.); mclund2@asu.edu (M.C.L.); simona.kraberger@asu.edu (S.P.K.); 2 School of Life Sciences, Arizona State University, Tempe, AZ 85287, USA; Martin.Wojciechowski@asu.edu; 3 Desert Botanical Garden, Phoenix, AZ 85008, USA; asalywon@dbg.org (A.M.S.); whodgson@dbg.org (W.C.H.); rpuente@dbg.org (R.P.-M.); 4 Florida Museum of Natural History, University of Florida, Gainesville, FL 32611, USA; lmajure@floridamuseum.ufl.edu; 5 The University of British Columbia, Vancouver, BC V6T 1Z4, Canada; 6 School of Behavioral and Brain Sciences, University of Texas at Dallas, 800 W Campbell Rd, Richardson, TX 75080, USA; 7 División de Biología Molecular, Instituto Potosino de Investigación Científica y Tecnológica, A.C., Camino a la Presa de San José 2055, Lomas 4ta Secc, San Luis Potosi, San Luis Potosí 78216, Mexico; jesus.avalos@ipicyt.edu.mx (J.A.A.-C.); grarguel@ipicyt.edu.mx (G.R.A.-A.); 8 Programa de Pós-Graduação em Botânica, Universidade Federal do Rio Grande do Sul, Porto Alegre-RS 90040-060, Brazil; matias.k@ufrgs.br; 9 UA Cancer Center, Department of Immunobiology, School of Animal and Comparative Biomedical Sciences, University of Arizona, Tucson, AZ 85721, USA; vandoorslaer@email.arizona.edu; 10 International Center for Agricultural Research in the Dry Areas (ICARDA), Terbol Station, Beqa’a, Zahle, Lebanon; s.kumari@cgiar.org; 11 Department of Integrative Biomedical Sciences, Computational Biology Division, Institute of Infectious Diseases and Molecular Medicine, University of Cape Town, Observatory, Cape Town 7925, South Africa; oyenirankehinde@gmail.com (K.A.O.); darrenpatrickmartin@gmail.com (D.P.M.); 12 CIRAD, UMR PHIM, 34090 Montpellier, France; christian.verniere@cirad.fr (C.V.); denis.filloux@cirad.fr (D.F.); philippe.roumagnac@cirad.fr (P.R.); 13 Plant Health Institute, University Montpellier, INRAE, CIRAD, 34090 Montpellier, France; 14 CIRAD, UMR PVBMT, F-97410 St. Pierre, France; pierre.lefeuvre@cirad.fr; 15 Embrapa Recursos Genéticos e Biotecnologia, Brasília 70770-917, Brazil; simone.ribeiro@embrapa.br; 16 Center for Evolution and Medicine, Arizona State University, Tempe, AZ 85287, USA; 17 Structural Biology Research Unit, Department of Clinical Laboratory Sciences, University of Cape Town, Cape Town 7925, South Africa

**Keywords:** Cactaceae, *Geminiviridae*, *Becurtovirus*, recombination, diversity

## Abstract

The family Cactaceae comprises a diverse group of typically succulent plants that are native to the American continent but have been introduced to nearly all other continents, predominantly for ornamental purposes. Despite their economic, cultural, and ecological importance, very little research has been conducted on the viral community that infects them. We previously identified a highly divergent geminivirus that is the first known to infect cacti. Recent research efforts in non-cultivated and asymptomatic plants have shown that the diversity of this viral family has been under-sampled. As a consequence, little is known about the effects and interactions of geminiviruses in many plants, such as cacti. With the objective to expand knowledge on the diversity of geminiviruses infecting cacti, we used previously acquired high-throughput sequencing results to search for viral sequences using BLASTx against a viral RefSeq protein database. We identified two additional sequences with similarity to geminiviruses, for which we designed abutting primers and recovered full-length genomes. From 42 cacti and five scale insects, we derived 42 complete genome sequences of a novel geminivirus species that we have tentatively named Opuntia virus 2 (OpV2) and 32 genomes of an Opuntia-infecting becurtovirus (which is a new strain of the spinach curly top Arizona virus species). Interspecies recombination analysis of the OpV2 group revealed several recombinant regions, in some cases spanning half of the genome. Phylogenetic analysis demonstrated that OpV2 is a novel geminivirus more closely related to viruses of the genus *Curtovirus*, which was further supported by the detection of three recombination events between curtoviruses and OpV2. Both OpV2 and Opuntia becurtoviruses were identified in mixed infections, which also included the previously characterized Opuntia virus 1. Viral quantification of the co-infected cactus plants compared with single infections did not show any clear trend in viral dynamics that might be associated with the mixed infections. Using experimental *Rhizobium*-mediated inoculations, we found that the initial accumulation of OpV2 is facilitated by co-infection with OpV1. This study shows that the diversity of geminiviruses that infect cacti is under-sampled and that cacti harbor diverse geminiviruses. The detection of the Opuntia becurtoviruses suggests spill-over events between viruses of cultivated species and native vegetation. The threat this poses to cacti needs to be further investigated.

## 1. Introduction

Plants in the family Cactaceae have adapted to a wide-range of environmental conditions, which is reflected in a remarkable diversity of growth forms [1,2]. They are characterized by having adapted to diverse edaphically arid and semi-arid environments and, with the exception of the species *Rhipsalis baccifera* (Sol.) Stearn, which naturally occurs in Madagascar and South Africa [3,4], are native to the Americas. One of the regions with the highest diversity and abundance of cacti is North American deserts, such as the Sonoran, Chihuahuan and Mojave deserts [5]. However, several species have been introduced to all continents except Antarctica [3], especially as ornamental plants. Cacti are important for the ecosystems in which they exist by providing shade, water and sources of food for a variety of animals. The cactus genus *Opuntia* (commonly referred to as prickly pears and nopales) is also economically and culturally relevant for certain communities since their fruits, and sometimes stem segments, are also used as food sources [6]. In addition, cacti are farmed for the breeding of cochineal insects (genus *Dactylopius*) that produce the carminic acid dye used as a colorant for food, textiles, and in the pharmaceutical industry [7].

The known diversity of viruses associated with cacti has mostly been restricted to RNA viruses belonging to the single-stranded RNA virus families *Alphaflexiviridae*, *Betaflexiviridae*, *Puribunyaviridae*, *Tombusviridae* and *Virgaviridae* [8,9,10,11,12,13,14], but through recent research efforts, DNA viruses that either infect or are associated with cacti have been identified [15,16,17,18].

The known plant-infecting DNA viruses are classified into three families: *Geminiviridae* [19], *Nanoviridae* [20] and *Caulimoviridae* [21]. Two caulimoviruses have been described as infecting the cactus *Epiphyllum* [17,18] and our group has identified a divergent geminivirus named Opuntia virus 1 (OpV1) found to infect several cactus species in the USA and Mexico [15]. These recent findings suggest that the diversity of DNA viruses that infect cacti have been seriously under-sampled and therefore highlight the importance of future research efforts in this area.

Members of the family Geminiviridae [19] are plant-infecting viruses that contain a circular single-stranded DNA genome encapsidated in twinned semi-icosahedral particles [22,23]. Currently, the family Geminiviridae is divided into nine genera: *Becurtovirus*, *Begomovirus*, *Capulavirus*, *Curtovirus*, *Eragrovirus*, *Grablovirus*, *Mastrevirus*, *Topocuvirus*, and *Turncurtovirus* [19,24]. There are also several highly divergent unclassified geminivirus species (*n* = 14) [15,25,26,27,28,29,30,31,32,33,34,35]. The International Committee on Taxonomy of Viruses (ICTV) is reviewing a proposal to accommodate some of these divergent species and create five new genera named *Citlodavirus*, *Maldovirus*, *Mulcrilevirus*, *Opunvirus* (to accommodate OpV1), and *Topileviru*s [36].

Geminiviruses are well known as causative agents of viral diseases in various crop plants and are associated with high yield losses [37,38]. However, many of the divergent members of the family (including most of the unclassified species) have been identified in non-cultivated native plants. In some instances, plants showed no observable visual symptoms, or only very mild symptoms caused by the virus [15,27,33,39,40,41]. The native and non-cultivated plants within which these divergent geminiviruses occur can act as reservoirs for viral species with the potential to emerge as crop pathogens [42,43].

Another area of concern is the far less well studied possibility of geminiviruses and other plant-infecting viruses spilling-over from agricultural crops to native vegetation. Studying such spillovers requires the study of viruses within an ecological framework, which some studies are beginning to address [44,45,46], and these are reviewed in [47,48]. In the case of plants such as cacti, which are often vegetatively propagated, the movement of infected plant material can mediate long-distance viral spreading [49]. This is especially likely to occur when the viruses concerned have no, or only very mild, associated symptoms. Although long-lived and/or vegetatively propagated plants are expected to select for viral variants with low levels of virulence, such plants can also be more susceptible to multiple or mixed infections, which can potentially select for increased virulence [50]. Indeed, geminiviruses that infect long-lived perennial plants are commonly found in mixed infections [51], which provides such viruses with more opportunities to recombine with members of other virus species.

Following the initial identification of OpV1, we decided to broaden the search for other DNA viruses infecting cacti in order to understand more about their viral community structure. Upon further investigation using high-throughput sequencing (HTS) data we identified fragments of sequences with similarity to other geminiviruses. Here we describe and characterize a novel divergent group of geminiviruses that we name Opuntia virus 2 (OpV2; 42 genomes), and a group of viruses (32 genomes) that fall within the genus *Becurtovirus* (sharing 82% identity with the member of *Spinach curly top Arizona virus* species). Although most of these viruses were found within single infections, in a few cases they were within detectably mixed infections, in some cases with OpV1 [15]. Infectivity assays were conducted with OpV2, the Opuntia becurtovirus, and OpV1 to evaluate viral load during co-infections, as well as to compare viral load between laboratory-infected *Nicotiana benthamiana* plants and naturally infected cactus samples.

## 2. Materials and Methods

### 2.1. Sample Collecting and Processing

Tissue samples were obtained from 577 plants in the Cactoideae and Opuntioideae clades [2]. These samples were collected from 19 countries: Argentina (*n* = 14), Bolivia (*n* = 8), Brazil (*n* = 8), Cuba (*n* = 1), Curaçao (*n* = 1), Dominican Republic (*n* = 2), France (*n* = 20), Haiti (*n* = 2), Lebanon (*n* = 1), Morocco (*n* = 1), Mexico (*n* = 31), Nigeria (*n* = 50), Paraguay (*n* = 3), Réunion (19), Spain (*n* = 6), Tunisia (*n* = 10), Uruguay (*n* = 5), the United States (*n* = 394), and Venezuela (*n* = 1) [15]. Samples were collected using either a 3-mm biopsy punch or scalpel blades and total DNA was extracted and processed as described previously [15]. In cases where plants were infested with cochineal insects, a cohort of about 5 –10 insects were collected and used for total DNA extraction, as described previously [15].

### 2.2. Geminivirus Genome Identification and Recovery

Using the previously acquired high-throughput data from cacti samples, the contigs were mined for those with similarities to geminiviruses [15]. Two contigs with detectable homology to known geminiviruses were identified. Pairs of abutting primers were designed (OpV2_F 5′-CAT GTA TTT CAT CAT TTA CAA AAA GCA GAC TTA-3′ and OpV2_R 5′-ATT TAG ATA TGG AGC AGA TTT GTT CCT CTT TT-3′; Bec_F 5′-TTG ATT TCG TTA GGC AAC CTA TTG AAT TCT-3′ and Bec_R 5′-AGA GTG GGC AGA ACA TAA TAT TTA TTT CGT-3′) to recover potentially full-length virus genomes from plant and insect samples. The primers were used to amplify the geminivirus genomes using KAPA HiFi HotStart DNA polymerase (KAPA Biosystems, Wilmington, MA, USA), following the thermal cycling protocol: initial denaturation at 95 °C for 3 min, followed by 25 cycles at 98 °C for 20 s, at 60 °C for 15 s, at 72 °C for 3 min, and a final elongation at 72 °C for 3 min and a final renaturation at 4 °C for 10 min. Amplicons with a size between 2.5 and 3.5 kb (the size range of geminiviruses genomes) were resolved in 0.7% agarose gels, excised, purified, and cloned in the pJET1.2 cloning vector (Thermo Fisher Scientific, Waltham, MA, USA). Cloned amplicons were Sanger sequenced by means of primer walking at Macrogen Inc. (South Korea). Genome assemblies and annotations were performed using Geneious 11.1.5 (Biomatters Ltd., Auckland, New Zealand).

### 2.3. Infectivity Assays

One isolate of each virus was used for the infectivity assays. The OpV1 isolate infectious clone construction has been previously described [15]. The OpV2 isolate DBG_56 (GenBank accession MT840871) was used for the infectious clone, and this genome was recovered from an *Opuntia basilaris* sample from the Desert Botanical Garden (Phoenix, AZ, USA) collected in 2018. The Opuntia-derived becurtovirus isolate S18_40 (GenBank accession MT840851) that was recovered from an *Opuntia aciculata* sample growing in an urban area in Tempe (Arizona, USA) in 2018 was used to construct the infectious clone. Specific primers were designed to amplify two copies of each Opuntia-derived geminivirus that were cloned in tandem to the binary vector pJL-89 [52], excluding the 35S promoter region of the vector, using the Gibson assembly [53] (New England Biolabs, Ipswich, MA, USA), as previously described by Ferro et al. [54]. Each clone was transformed into competent *Escherichia coli* XL1 Blue cells, and to confirm that the ligation occurred correctly, clones were analyzed by digestion with EcoRV (OpV2) and NdeI/SalI (becurtovirus). The clone containing the two tandemly cloned copies of the Opuntia-derived geminiviruses were then used to transform *Rhizobium radiobacter* (synonymous species name for *Agrobacterium tumefaciens*) GV3101.

Initial infection assays were performed in *N. benthamiana* plants that were inoculated with OpV2 and the Opuntia becurtovirus as single infections. Furthermore, to evaluate mixed infection dynamics, *N. benthamiana* plants were inoculated with both OpV1/OpV2, OpV1/Opuntia becurtovirus, or OpV2/Opuntia becurtovirus to mirror mixed infections found in cactus plants. In all *Rhizobium*-inoculations, *R. radiobacter* was grown for 20 h in Luria broth with kanamycin (50 g/mL) and rifampicin (50 g/mL). The culture was then centrifuged for 10 min at 4600 rpm to pellet the cells before resuspension in MES buffer (10 mM MES hydrate and 10 mM MgSO4•7H_2_O) with acetosyringone 150 μM to an OD of 1.0. In the mixed infection, equal volumes of both viruses (OD 1.0) were mixed together prior to inoculation.

### 2.4. Southern Blot Analysis

Southern blots were used to detect the replicative forms of OpV2 during infection. Total nucleic acid was extracted from inoculated *N. benthamiana* plants as described above. Five µg total DNA from each plant, along with a positive control (5 ng of OpV2 PCR amplified genome), and one negative control of an uninoculated *N. benthamiana* plant, were resolved in a 1% agarose gel. The nucleic acid from the gel was transferred to a positively charged Hybond-N+ nylon membrane (GE Healthcare, Chicago, IL, USA) and UV-crosslinked using the default setting of the UV Stratalinker 1800 (Stratagene, La Jolla, CA, USA). The membrane was hybridized with a digoxigenin-labeled probe of the full-length OpV2 genome. The hybridization and probe preparation were carried out using a DIG High Prime DNA Labeling and Detection Starter Kit I (Roche, Basel, Switzerland), as directed by the manufacturer.

### 2.5. Viral Particle Purification and Transmission Electron Microscopy

Approximately 100 g of *N. benthamiana* leaf material 45 days post-inoculation with the OpV2 infectious clone (GenBank accession MT840871) was homogenized in 100 mL of extraction buffer (1 × PBS, 10 mg/mL sodium ascorbate, 2 mM PMSF, 1 mM EDTA). The homogenate was filtered through two layers of miracloth and two layers of cheesecloth, and subsequently centrifuged three times for 30 min at 14,800 rpm until clear. The clear homogenate was centrifuged for 4 h at 32,000 rpm using a Beckman 32 Ti rotor (Beckman, USA) onto a 10% sucrose cushion, and the pellet was resuspended in 1 mL of 1× PBS. Ten microliters of the resuspended pellet was absorbed onto carbon-coated copper grids for 2 min, washed three times, and stained with 2% uranyl acetate. The grids were viewed using a Philips CM 200 transmission electron microscope (Philips, Amsterdam, The Netherlands).

### 2.6. Viral Load Quantification

Absolute viral quantification was performed by means of real-time quantitative PCR (qPCR). Reactions were performed with either 40 ng of total *N. benthamiana* DNA or 10 ng of infected cactus plant DNA in a 10-µL qPCR reaction with 2x iTaq Universal SYBR Green Supermix (Bio-rad, USA), and 500 nM of each primer pair (Supplementary Table S1). Standard curves (StC) for each virus were prepared using a tenfold serial dilution of the plasmid containing the viral full-length genome diluted in 2.5 ng/µL of total DNA extracted from uninoculated *N. benthamiana* plants. The dilutions ranged from 10^8^ to 10 copies of viral DNA (genomic units) per microliter. All qPCR experiments contained a negative control and a non-template control, and were performed in triplicate. Reactions were performed in a Bio-rad CFX96 Real-time PCR System with the following conditions: 95 °C for 3 min, followed by 40 cycles of 95 °C for 30 s and 60 °C (varied by primer, Supplementary Table S1) for 30 s. A final melting curve analysis was performed ranging from 65 °C to 95 °C, with 0.5 °C increments every 5 s.

### 2.7. Pairwise Identity and Phylogenetic Analyses

Genome-wide pairwise identity comparison of the 32 cactus-derived becurtoviruses and the 42 OpV2 sequences were performed using SDT v.1.2 [55].

The full-length nucleotide sequences of representatives from each genotype (OpV2 and becurtovirus), together with those encoded by representatives of each geminivirus genera, including currently unclassified geminiviruses (*n* = 49), were aligned using MAFFT v.7 [56]. The resulting alignment was used to infer a neighbor-joining phylogenetic tree using the Jukes–Cantor nucleotide substitution model with 1000 bootstrap replicates to test branch support. Branches with <60% bootstrap support were collapsed using TreeGraph2 [57] and the tree was midpoint rooted.

In addition, the full-length nucleotide sequences of the OpV2 group (*n* = 42) and becurtovirus (*n* = 32) were each aligned using MUSCLE [58]. Each alignment was used to infer a neighbor-joining tree using the Jukes-Cantor substitution model with 1000 bootstrap replicates to test branch support. Branches with <60% bootstrap branch support were collapsed using TreeGraph2 [57]. The 42 and 32 genomes sequences, respectively, of the group OpV2 and cactus-derived becurtovirus, with fragments derived through inter-species recombination removed (see below), were used to infer a maximum-likelihood (ML) phylogenetic tree using IQ-TREE [59]. The model of nucleotide substitution used for the OpV2 tree was GTR+F+I+G4 and for the cactus-derived becurtovirus was TIM2+F+I+G4, as determined by ModelFinder [60] with 1000 bootstrap replicates to test for branch support.

Datasets were assembled for the inferred replication-associated protein (Rep) and capsid protein (CP) amino acid sequences from representatives of the OpV2 and becurtovirus genotype groups, along with representative sequences from the geminivirus genera and unclassified geminiviruses (*n* = 49). The Rep and CP amino acid sequence datasets were aligned using MAFFT v.7 [56], and the alignments were used to infer ML phylogenetic trees with models rtRev+G+I for the CP data and rtRev+G+I+F for the Rep data, as determined to be best-fitting by ProtTest [61] and with the approximate maximum likelihood ratio test (aLRT) of branch support. Branches with <80% aLRT support were collapsed using TreeGraph2 [57], and each ML tree was rooted with inferred Rep or CP amino acid sequences from two members of the viral family Genomoviridae [62].

### 2.8. Recombination Analyses

The 42 OpV2 genomes and 32 becurtovirus genome sequences were aligned using MUSCLE [58], and the resulting alignments were used for intra-species recombination analysis using RDP v.5.5 [63] with default settings using the detection methods RDP [64], GENECONV [65], BOOTSCAN [66], MAXCHI [67], CHIMERA [68], SISCAN [69], and 3SEQ [70]. Only recombination events that were detected by more than three methods with a *p*-value <0.05 were accepted.

Based on the ML phylogeny of the encoded Rep and CP amino acid sequences, we also decided to detect recombination events at an inter-generic level using representative sequences of the *Becurtovirus* and *Curtovirus* genera, together with one representative genome from OpV2, and the cactus-derived becurtovirus sequences were aligned using MUSCLE [58]. The nucleotide sequences on either side of the Rep and CP coding regions were removed from the alignment (these regions are non-homologous between the groups and can therefore not be aligned in any meaningful way), and this was then used for recombination analysis using RDP v5.5 [63] with the same standards used for the inter-species analysis.

### 2.9. Capsid Protein Cluster Analysis

The CP amino acid sequences of all geminiviruses available in GenBank were extracted and a representative dataset was generated through cluster analysis, using CD-HIT [71] with a 90% identity threshold. One representative from each cluster, together with representatives of the 11 genotypes of OpV2, 15 genotypes of OpV1 and one representative from the Opuntia becurtoviruses were used to generate a sequence similarity network using the enzyme function initiative–enzyme similarity tool (EFI-EST) [72] with a similarity score of 65 and an E-value threshold of 1 × 10^−5^. The network was visualized in Cytoscape v3.8.2 [73] with the organic layout.

## 3. Results and Discussion

As a result of the use of HTS technologies, there has been a steady increase in the number of divergent geminiviruses that have been discovered. These newly discovered viruses have further illuminated the vast breadth of geminivirus diversity and have revealed the complex evolutionary history of this family. The combination of HTS with the rolling circle amplification technique has helped to facilitate the identification of geminiviruses without prior knowledge of the circulating viral population. Additionally, research efforts, which have been expanded to include uncultivated native plant species instead of exclusively crop plants displaying visible disease, have contributed tremendously to revealing the true evolutionary and ecological contexts of the known geminivirus crop pathogens. As a consequence, in the past five years, four new genera within the family Geminiviridae have been recognized [24,74] to accommodate the novel divergent viruses identified through HTS technologies, whereas an additional five new genera are currently under consideration by the ICTV [36]. These findings demonstrate that the diversity of plant-infecting viruses associated with crop diseases only represent a small proportion of the global plant-virus diversity [75]. The study of geminivirus diversity will be of the utmost importance in the context of agro-ecological interfaces [42,44,76,77], where spill-over between agricultural and native vegetation is most likely to occur, and where these spill-overs are likely to have the most significant ecological and/or agricultural impact [78,79]. Particularly in areas where the environment has been anthropogenically modified, any sudden changes in host population structure and viral transmission can lead to viral population changes and possible disease emergence [80]. For long-lived and vegetatively propagated plants with little industry, such as cacti, no sanitation measures exist and the management of infected plant material can be difficult, especially when pathogens have low virulence and plants do not display apparent symptoms.

### 3.1. Geminiviruses Infecting Cacti

In an initial analysis of cactus samples collected from 18 countries, we identified a novel geminivirus named OpV1 [15]. That initial finding led us to take a deeper look into the HTS data obtained from the cactus plants in this study, and consequently two other sequence fragments with similarity to geminiviruses were identified. By using two pairs of abutting primers, amplicons of ~3 kb were amplified from the cactus samples in question.

Sequence analyses of the putative full-length genomes revealed a divergent geminivirus, which we have tentatively named Opuntia virus 2 (OpV2), and a new becurtovirus strain of the spinach curly top Arizona virus species that we refer to as Opuntia becurtovirus (Table 1).

### 3.2. Opuntia Virus 2

A total of 42 OpV2 genome sequences were recovered from 16 cactus plants and three cochineal insects collected in 2018 from the USA. With the exception of one plant sample from Utah, the remaining samples were collected in the state of Arizona (Table 1). Additionally, two of the groups of cochineal insects from which OpV2 genomes were recovered were collected from OpV2-positive plants (Table 1). None of the plants presented any apparent symptoms.

#### 3.2.1. Genome Organization and Diversity

**Figure 1:**
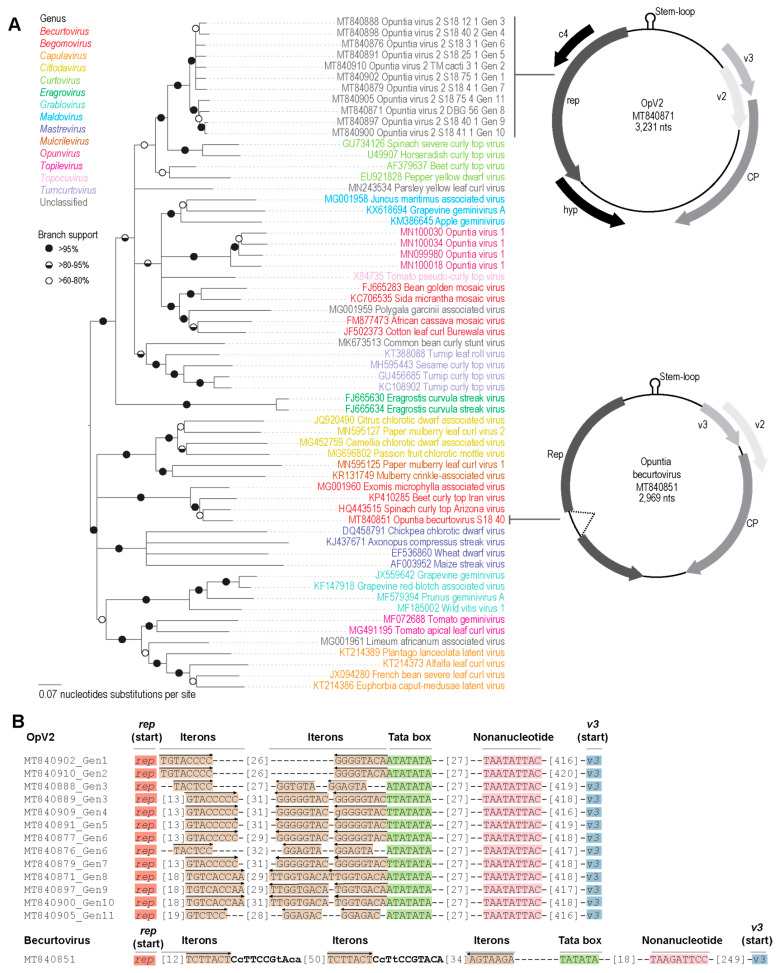
(**A**) Neighbor-joining phylogenetic tree of the genome sequences of the eleven genotypes of OpV2 and the Opuntia becurtovirus, together with representative sequences from various genera in the family *Geminiviridae*. Branches with <60% bootstrap support have been collapsed and the phylogenetic tree is midpoint-rooted. The genomic organization of OpV2 and the Opuntia becurtovirus are illustrated on the right side of the phylogenetic tree near their respective groups. (**B**) Organization and orientation of the replication-associated interactive sequences “iterons” in the intergenic region of both eleven genotypes of OpV2 and the Opuntia becurtovirus. The arrows indicate the orientation of the iteron sequences relative to the nonanucleotide and lower-case letters indicate that the nucleotide is variable among sequences in that genotype. Some genotypes presented more than one type of “iteron” sequence.

The OpV2 sequences recovered ranged from 3194–3247 nt in size and have a genomic organization similar to other geminiviruses, containing at least six recognizable open-reading frames (ORFs) encoding proteins >84 amino acids. Interestingly, the OpV2 genome size is about 10% larger than most other known geminiviruses; however, other divergent geminiviruses recently identified through HTS approaches also present genomes > 3000 nt [26,28,32]. Based on sequence similarity with geminivirus-expressed proteins, the OpV2 ORF’s in the complementary strand likely encode a replication-associated protein (Rep), a symptom determinant protein (C4), and a hypothetical protein. Additionally, the OpV2 virion strand likely encodes a capsid protein (CP), a regulatory protein (V2), and a possible movement protein (V3) (Figure 1). The 42 isolated genomes from OpV2 share 90.3%–100% identity to each other and were grouped into 11 genotypes, based on a 95% nucleotide identity cut-off (Table 1; Supplementary Data S1). Interestingly, two of the 16 plants analyzed were infected with more than two genotypes (Table 1).

All the OpV2 genomes contained the conserved geminivirus virion-strand origin of replication nonanucleotide sequence “TAATATTAC”, which is located within a sequence that is capable of forming a hairpin-loop structure with the conserved nonanucleotide in its loop. In the long intergenic region, we also identified iterative potential replication-associated protein recognition sequences, commonly referred to as “iterons,” and a potential TATA box (Figure 1). Among the OpV2 genotypes two “iterons” were identified, one adjacent to the TATA box and the other immediately upstream of the Rep gene. It is interesting to note that in eight of the eleven OpV2 genotypes, the “iteron” adjacent to the TATA box occurs as two tandem TATA repeats. Sequences from genotypes 3 and 6 present two different “iteron” sequences.

The OpV2 representative genotypes all share <74.1% nucleotide pairwise identity with other geminiviruses (Supplementary Data S1). Their encoded Rep and CP proteins respectively share <76.8% and <85.4% amino acid sequence identity with other geminiviruses (Supplementary Data S2). Hence, the OpV2 sequences are diverse compared to the currently classified geminiviruses and likely represent a new genus. The Rep amino acid sequences encoded by OpV2 all have a rolling-circle replication, geminivirus Rep sequence (GRS), a helicase SF3, and Walker motifs [81], which are other important conserved features shared with geminiviruses and which are essential for the rolling circle replication functions of Rep (Supplementary Table S2).

**Figure 2:**
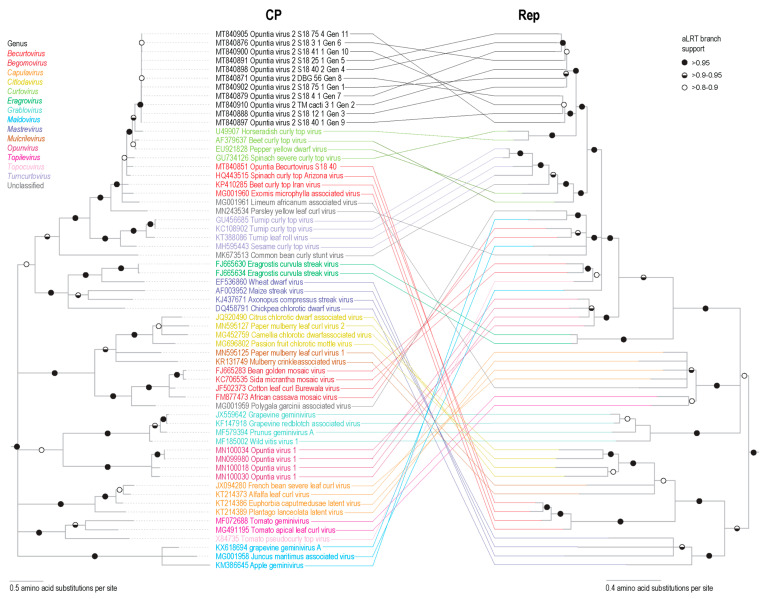
Maximum-likelihood phylogenetic tree of the Rep and CP amino acid sequences of the eleven representative genotypes of OpV2 and Opuntia becurtovirus, and representative sequences from various genera in the family *Geminiviridae*. Branches with <0.8 aLRT support have been collapsed and the trees are rooted with sequences of genomoviruses [62].

Phylogenetic analysis of representative OpV2 sequences, together with representatives of other geminiviruses (Figure 1), show that OpV2 clusters evolutionarily together with species in the genus *Curtovirus*. Not surprisingly, the highest genome-wide pairwise identities of the OpV2 sequences are with the members of the curtovirus species, such as that of beet curly top virus (AF379637) (Supplementary Data S2). In contrast, ML phylogenetic analysis of representative amino acid sequences of the encoded OpV2 Rep and CP proteins, together with those of other geminiviruses, demonstrate a more complex scenario. The OpV2 encoded CPs cluster with those of the curtoviruses, becurtoviruses, and two presently unclassified divergent geminiviruses, *Limeum africanum*-associated virus [31] and parsley yellow leaf curl virus [35] (Figure 2). The OpV2 encoded Rep sequences group together with those of two curtoviruses, horseradish curly top virus and spinach severe curly top virus (Figure 2).

#### 3.2.2. Identification of Recombination in OpV2

**Figure 3:**
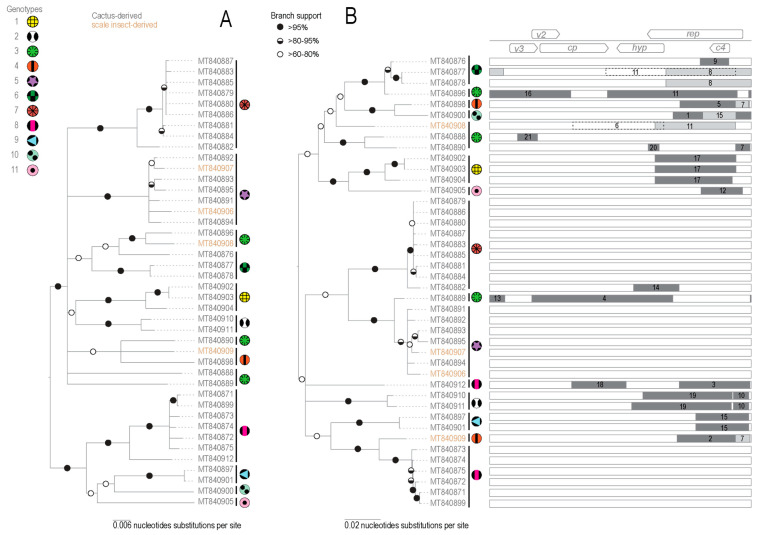
(**A**) Neighbor-joining phylogenetic tree of the 42 OpV2 sequences with genotypes denoted by symbols. (**B**) Maximum-likelihood phylogenetic tree of the 42 OpV2 sequences with recombination regions removed. The eleven genotypes of OpV2 are represented by symbols. Graphical representation of each genome representing the recombination event with the breakpoint location within the genome.

Recombination plays an important role in the evolution of geminiviruses [82,83,84]. We analyzed the 42 OpV2 sequences for evidence of recombination using RDP5 [63] and identified 21 well-supported recombination events (Figure 3, Table 2). Interestingly, genotypes 5 and 7 had no detectable recombinant regions. In genotype 8, only one of the seven sequences that comprise this group had detectable evidence of recombination. Recombinant region sizes ranged from 142 to 1790 nt. One sequence from genotype 3 had the largest recombination transferred genome fragment, which corresponds to nearly half of the genome, spanning the *cp* gene, small intergenic region, and a gene encoding a hypothetical protein (Figure 3).

The majority of the recombination events occur in the virion-strand gene coding regions. Additionally, the breakpoints for several recombination events are located in the long intergenic region where the origin of replication is located, a feature that has been extensively reported for other geminiviruses [15,43,84,85,86]. Collectively, the same recombination event seemed to occur in only one or up to three sequences and always within the same genotype, which indicates that the detected events likely occurred more recently than when the last common ancestor of the 42 OpV2 sequences existed. This demonstrates that coinfection between these viruses are common.

#### 3.2.3. Infection Assays

To assess the infectivity of OpV2, an infectious clone with two tandem copies of OpV2 DBG_56 (GenBank accession MT840871) was constructed. The infectivity assays in *N. benthamiana* plants showed that based on three independent assays performed with 10 inoculated plants in each, OpV2 had a 40–50% rate of systemic infection. OpV2 infectivity was further confirmed with Southern blot analysis of DNA from *N. benthamiana*-infected plants that revealed evidence of circular covalently closed DNA, super coiled dsDNA and single-strand DNA. No replicative forms of OpV2 were observed for the uninoculated control plants (Supplementary Figure S1A).

Transmission electron microscopy analysis of OpV2 particles recovered from inoculated *N. benthamiana* plant material revealed the presence of twinned icosahedral particles (Supplementary Figure S1B). This suggests that despite the fact that the OpV2 genome is approximately 10% larger than the most other characterized geminiviruses, the ~3.2 kb genome is still packaged into geminate particles.

### 3.3. Opuntia Becurtovirus

The *Becurtovirus* genus is currently composed of three species, beet curly top Iran virus, spinach curly top Arizona virus, and *Exomis microphylla* latent virus. Their genomic organization consists of a capsid protein gene (*cp*), a regulatory protein gene (*v2*) and a movement protein gene (*v3*) on the virion-sense strand and on the complementary-sense strand, a replication-associated protein gene (*rep*), potentially from alternatively spliced transcripts. The nonanucleotide “TAATATTAC” sequence that is found in the virion-strand origin of replication is highly conserved among the geminiviruses; however, viruses in the genus *Becurtovirus* have a different nonanucleotide sequence, i.e., “TAAGATTCC”. Thus far, becurtoviruses have been found to infect eudicotyledonous plants of the species *Beta vulgaris* (beet), *Beta vulgaris* subsp. *maritima* (beet), *Capsicum frutescens* (pepper), *Exomis microphylla*, *Phaseolus vulgaris* (bean), *Spinacia oleracea* (spinach), *Solanum lycopersicum* (tomato), *Solanum melongena* (eggplant) and *Vigna unguiculata* (black-eyed pea) [31,87,88,89,90,91,92,93,94].

Beet curly top Iran virus is transmitted by the leafhopper *Circulifer haematoceps*, a species commonly found in Iran [92]. The only becurtovirus previously described in the Americas is spinach curly top Arizona virus, a virus found in symptomatic spinach plants from Arizona, USA, in 2009 [93]. Since then, to our knowledge this becurtovirus has not been reported, likely due to under-sampling. The identification of the Opuntia becurtovirus could suggest a spill-over event in agro-ecological interface areas.

#### 3.3.1. Diversity of Opuntia Becurtoviruses

The becurtoviruses identified in the cactus samples share ~82% genome-wide pairwise identity with spinach curly top Arizona virus (Supplementary Data S2). According to the current species and strain demarcation for the genus *Becurtovirus* [74], the cactus-derived becurtovirus is a new strain from the species spinach curly top Arizona virus, which we have named Opuntia becurtovirus.

A total of 32 genome sequences of the Opuntia becurtovirus were obtained from 26 cactus plants and two cochineal insects collected in 2018 in the USA in the states of Arizona (*n* = 26), Texas (*n* = 1) and Utah (*n* = 1) (Table 1). The cactus-derived becurtovirus genomes range from 2899 to 2982 nt and have a similar genome organization as compared to other becurtoviruses (Figure 1). Based on the pairwise sequence identity results, the 32 genomes of cactus-infecting becurtovirus range from 94.8%–100% sequence identity among themselves (Supplementary Data S3) and based on a 95% identity cut-off they are all members of the same genotype. SCTAV is therefore tentatively assigned to genotype 1 and the Opuntia becurtovirus to genotype 2.

Phylogenetic analysis of the genome sequences from the Opuntia becurtoviruses with representatives of the geminivirus family shows, as expected, that they group with other becurtoviruses, being more closely related to SCTAV (Figure 1). The same is observed in the ML phylogenetic tree of the Opuntia becurtovirus encoded Rep and CP amino acid sequences (Figure 2). The rolling circle amplification GRS, helicase SF3 and Walker motifs [81] were also present in the predicted Rep amino acid sequences of the Opuntia becurtoviruses and all were very similar to their counterparts in the SCTAV isolates (Supplementary Table S2).

#### 3.3.2. Identification of Recombination in Becurtoviruses

**Figure 4:**
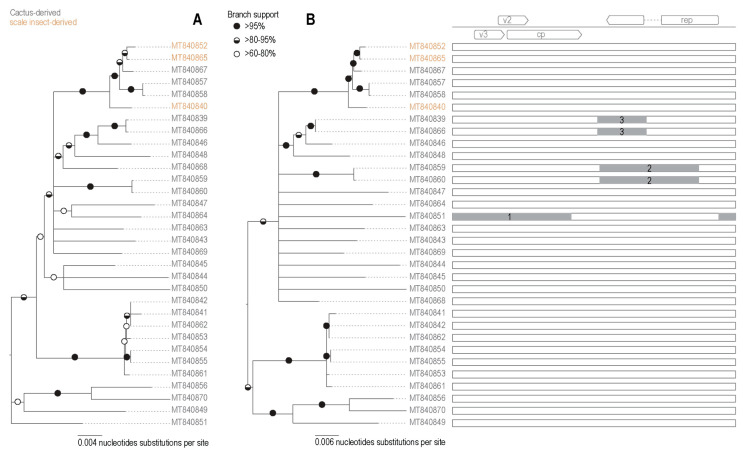
(**A**) Neighbor-joining phylogenetic tree of the 32 Opuntia becurtovirus sequences. Branches with <60% bootstrap branch support have been collapsed and the tree is midpoint-rooted. (**B**) Maximum-likelihood phylogenetic tree of the 32 Opuntia becurtovirus sequences with recombination regions removed. Graphical representation of each genome representing the recombination event with the breakpoint location within the genome.

In the interspecies recombination analysis of the Opuntia becurtoviruses, three well supported events were detected (Figure 4; Table 3). The recombinant regions ranged from 522 to 1640 nt. Again, the largest of the detected events (event 1 in Table 1) implied the recombination transfer of nearly half of the genome, spanning the intergenic region and the complementary sense-encoded proteins. Another event (number 2 in Table 1) was evident in two sequences, both derived from the same cactus sample (Table 1).

#### 3.3.3. Infectivity Assays

To evaluate the infectivity of the Opuntia becurtovirus, an infectious clone was constructed and used to inoculate *N. benthamiana* plants. In three independent experiments with 10 plants each, only two plants were detectably infected through conventional PCR at 45 days post-inoculation. We were not able to detect the replicative forms of the Opuntia becurtovirus after Southern blot analysis of the infected plants (data not shown). However, qPCR quantification of both *N. benthamiana* plants inoculated with Opuntia becurtovirus showed their presence at very low levels with approximately 100 viral copies in 40 ng of plant material or 2 to 3 copies/ng of plant material (Supplementary Figure S2). It is likely that Opuntia becurtovirus does not replicate well in *N. benthamiana* plants or a longer period of viral infection might be required to establish infection.

### 3.4. Inter-Genus Recombination

Viruses in several geminivirus genera show clear evidence of inter-genus recombination [39,77,93,95,96]. The geminivirus genomic organization, which involves bi-directionally transcribed genes and the geminivirus mode of rolling circle replication, are both factors that seem to influence the rates and patterns of recombination in these viruses [43,84,97].

Recombination is clearly evident in the incongruence observed in the phylogenetic trees of the encoded Rep and CP amino acid sequences (Figure 2). Since the OpV2 encoded Rep and CP protein sequences cluster together with those of viruses in the genera *Becurtovirus* and *Curtovirus*, we undertook a recombination analysis to detect any events of recombination between members of these genera. We took representatives of each genus, including one sequence from OpV2 and the Opuntia becurtovirus to create a dataset. The dataset sequences were aligned and this alignment was trimmed to retain only the nucleotide sequences of the Rep and CP coding regions (the only parts of the genomes that were clearly homologous).

**Figure 5:**
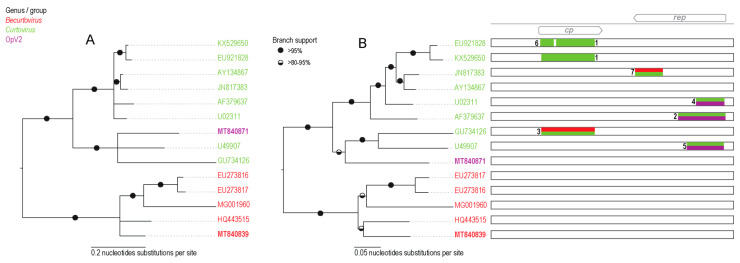
(**A**) Neighbor-joining phylogenetic tree of the OpV2 and becurtovirus representative sequences, together with representatives from the genera *Becurtovirus* and *Curtovirus*. Branches with <60% bootstrap branch support have been collapsed and the tree is midpoint-rooted. (**B**) Maximum-likelihood (ML) phylogenetic tree of the Opv2 and becurtovirus representative sequences, together with representatives from the genera *Becurtovirus* and *Curtovirus* with recombination regions removed. Branches with <60% bootstrap support have been collapsed and the tree is midpoint-rooted. Graphical representation of each genome, representing the recombination event with the breakpoint location within the OpV2 genome for reference, which is color coded according to the genera of the major and minor parent sequences of each event. In bold are the two sequences that represent OpV2 and Opuntia becurtovirus.

In the recombination analysis, seven well supported events were identified, all of them in the sequences of curtoviruses (Table 4, Figure 5). Three events were identified in the CP sequence of curtoviruses, two of which were interspecies events. In one event (event 3 in Figure 5), the recombinant region appears to be derived from curtoviruses and becurtoviruses. Recombination events 1 and 3 span almost the entire CP nucleotide sequence. In the Rep coding region, four events were detected. One displays contributions from both curtoviruses and becurtoviruses and the other three are recombinants between curtoviruses and OpV2 (Figure 5).

### 3.5. Mixed Infections of Geminiviruses in Cacti

Geminivirus mixed infections are frequently found in nature. There are several records of mixed infection from species in the same genera, especially species in the genus *Begomovirus* [85,98,99,100]. In some cases, co-infecting viruses can have synergistic interactions, leading to increases in pathogenicity [101,102,103,104,105]. Mixed infections also allow for the exchange of genetic material through recombination [65,86], which can lead to the emergence of new variants, species, and genera.

**Figure 6:**
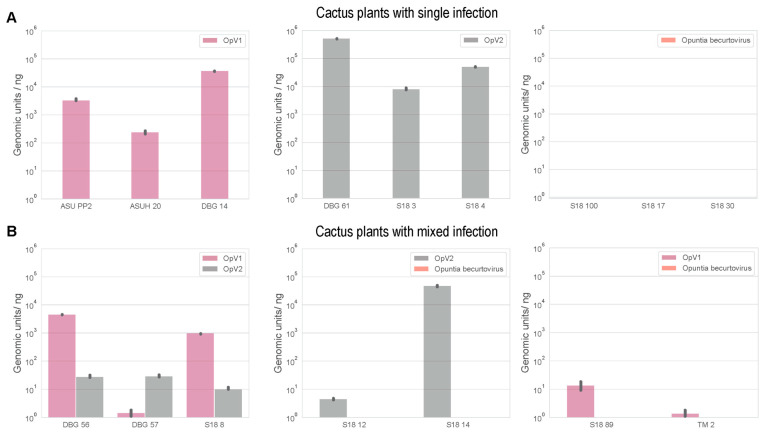
Bar graph of the viral loads (genomic units/ng of total DNA) determined by real-time quantitative PCR for the Opuntia virus 1 (OpV1), Opuntia virus 2 (OpV2), and Opuntia becurtovirus in a subset of cactus samples collected in this study. The graphs show (**A**) the group of single-infected cacti and (**B**) the cactus samples that presented mixed infection with standard deviation for the triplicate reactions. The Opuntia becurtovirus was the only virus that did not present detectable levels of genomic units.

Naturally occurring mixed infections were detected in a few cactus samples in our study (Table 1). Two plants were infected with both OpV1 and OpV2, whereas Opuntia becurtovirus was also found in co-infections with OpV1 (*n* = 6) and OpV2 (*n* = 2). We used qPCR to quantify the viral loads of these co-infected plants compared to single infections in other cactus samples. Unsurprisingly, Opuntia becurtovirus was not detected by qPCR in either the co-infected or single infected *N. benthamiana* plants, which means the virus in these plants was at very low titers, since we used 10 ng of DNA from a total plant DNA extract for the quantification reactions. Nevertheless, both OpV1 and OpV2 were detected by means of qPCR, and cactus samples had varying viral loads (genomic units/ng) (Figure 6). There is no clear evidence in the cactus samples that co-infection seems to be favoring one virus over the other (Figure 6). It should be noted although the cacti analyzed are different species (Table 1), which might help explain the variable qPCR results. However, there were also variations in viral load within the same cactus plants at different locations within the plants. In the *Opuntia basilaris* plants co-infected with OpV1 and OpV2, two pads from the same plant were collected (DBG_56 and DBG_57) and showed different co-infection dynamics. In one pad (DBG_56), OpV1 had a higher viral load than OpV2, whereas in the second pad (DBG_57), OpV2 had a higher viral load than OpV1 (Figure 6). It is unknown if geminiviruses can establish a systemic infection in cacti, and further research is certainly needed to address this.

**Figure 7:**
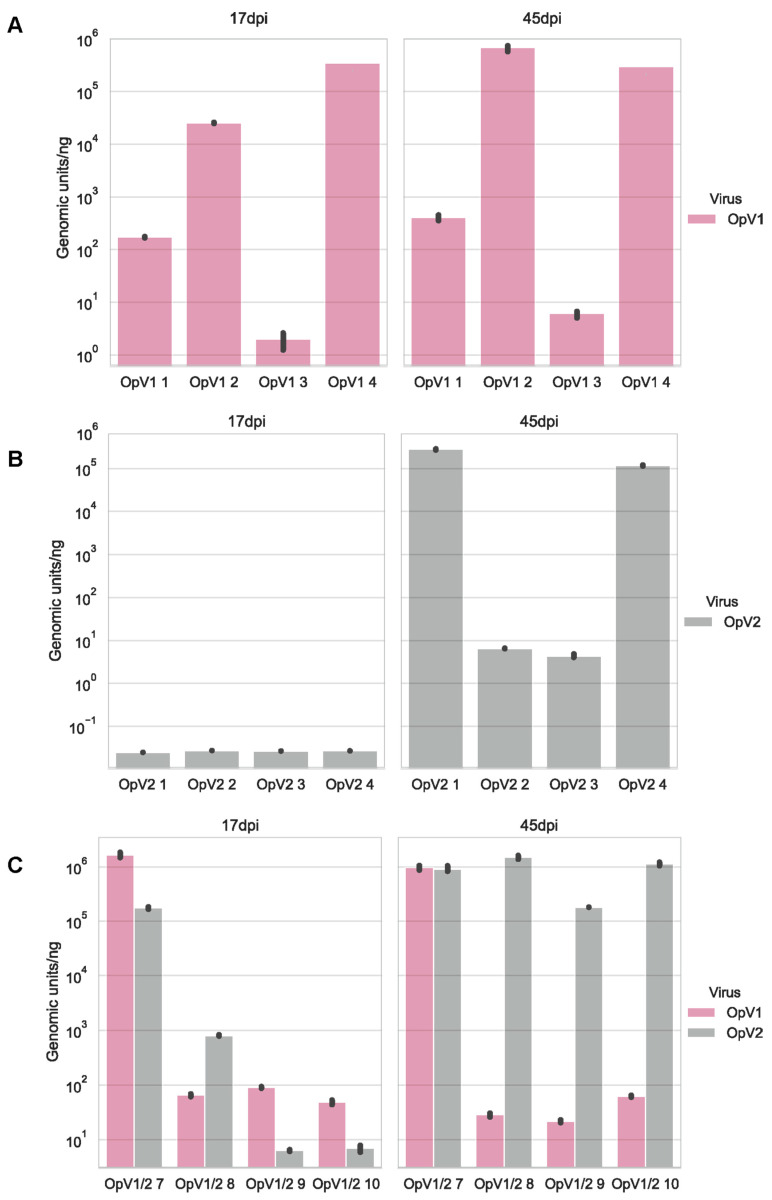
Bar graph of the viral loads (genomic units/ng total DNA) observed through real-time quantitative PCR for the Opuntia virus 1 (OpV1) and Opuntia virus 2 (OpV2) in *N. benthamiana* plants at 17 and 45 days post-inoculation. (**A**) *N. benthamiana* inoculated with OpV1; (**B**) *N. benthamiana* inoculated with OpV2 and (**C**) *N. benthamiana* plants with mixed infection of OpV1 and OpV2 with standard deviation for the triplicate reactions.

To further investigate the potential dynamics of those co-infections, infectivity assays were performed with OpV1 and OpV2 in *N. benthamiana*. Of 10 co-inoculated *N. benthamiana* plants, four were found to be co-infected, one plant was found to be infected with only OpV2, and five plants only with OpV1. The four plants with mixed infections and four of the plants with single infections were used for viral load quantification using qPCR. All the samples were analyzed at two time points, 17- and 45-days post-inoculation (dpi). OpV1 infected plants showed little difference in viral load between the time points (Figure 7). However, OpV2 could only be detected at 45 dpi but with similar viral loads to plants with OpV1 infections (Figure 7). The mixed-infection plants showed some interesting dynamics. At 17 dpi OpV2 was detectable, and its viral load significantly increased by 45 dpi (Figure 7). On the other hand, OpV1 maintained similar viral loads over the two time points. These results suggests that OpV2 favors mixed infections with OpV1, but in this case it did not seem to affect the viral load of OpV1.

In attempted co-infections with OpV1 and Opuntia becurtovirus and OpV2 and Opuntia becurtovirus, the fact that Opuntia becurtovirus was not detected was not surprising based on the initial single infection experiments. Therefore, we could not draw any conclusions on coinfections involving this species (data not shown).

### 3.6. Cluster Analysis of Geminivirus Capsid Protein

**Figure 8:**
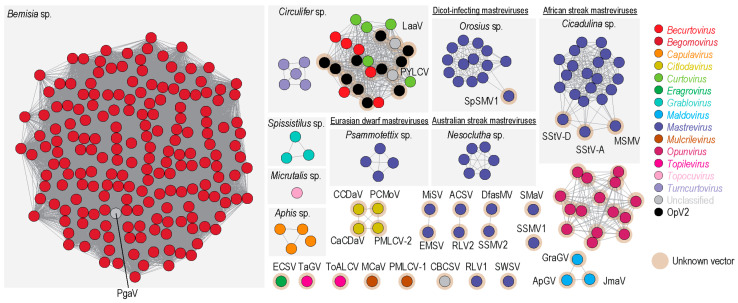
Sequence similarity network analysis of the CP amino acid sequences of geminiviruses present in GenBank (dataset was created with an amino acid identity cut-off of 90%), together with representatives of the 11 genotypes from OpV2, 15 genotypes from OpV1, and one Opuntia becurtovirus. Each dot represents a sequence and is colored based on the genus or group. The genera that have known insect vectors are highlighted in a light grey box with the insect vector name displayed at the top. Clusters or singletons marked with a brown halo have no known insect vector associated with them. ApGV, apple geminivirus; ACSV, *Axonopus compressus* streak virus; CaCDaV, Camellia chlorotic dwarf-associated virus; CCDaV, citrus chlorotic dwarf-associated virus; DfasMV, dragonfly-associated mastrevirus; CBCSV, common bean curly stunt virus; ECSV, *Eragrostis curvula* streak virus; EMSV, *Eragrostis minor* streak virus; GraGV, grapevine geminivirus; JmaV, *Juncus maritimus*-associated virus; LaaV, *Limeum africanum*-associated virus; MCaV, mulberry crinkle- associated virus; MiSV, *Miscanthus* streak virus; MSMV, maize streak Reunion virus; PYLCV, parsley yellow leaf curl virus; PMLCV-1, paper mulberry leaf curl virus 1; PMLCV-1, paper mulberry leaf curl virus 2; PCMoV, passion fruit chlorotic mottle virus; PgaV, *Polygala garcinii*-associated virus; RLV1, rice latent virus 1; RLV2, rice latent virus 2; SMaV, switchgrass mosaic-associated virus; SpSMV1, sweet potato symptomless mastrevirus 1; SSMV1, *Sporobolus* striate mosaic virus 1; SSMV2, *Sporobolus* striate mosaic virus 2; SStV-A, sugarcane striate virus A; SStV-D, sugarcane striate virus D; SWSV, sugarcane white streak virus; TaGV, tomato-associated geminivirus; ToALCV, tomato apical leaf curl virus.

Geminivirus CP amino acid sequences are possibly co-diverging with their specific insect vectors, as highlighted in the phylogenetic analysis presented in [48]. Hence, we undertook a sequence similarity network analysis of the CP amino acid sequences of all geminiviruses (with a >90% identity cut off) and further associated the identified clusters with the known insect vectors (Figure 8). Sequence similarity network analyses are useful for clustering groups of similar protein sequences in large datasets relatively quickly (see [48,106,107,108] for examples). Unlike OpV1, which forms its own distinct cluster [15], indicating that it is likely transmitted by an insect vector that has not yet been associated with geminiviruses, OpV2 CPs cluster with those of curtoviruses, becurtoviruses (including Opuntia becurtovirus), and two unclassified viruses (parsley yellow leaf curl virus and *Limeum africanum*-associated virus). Becurtoviruses and curtoviruses are known to be transmitted by leafhoppers (*Circulifer* sp.), and based on the cluster analysis, it is highly likely that OpV2 is also transmitted by this insect vector. Controlled insect transmission experiments will be necessary to properly test this hypothesis.

The identification of OpV2 and Opuntia becurtoviruses expands the diversity of known geminiviruses that are associated with plants in the family Cactaceae. OpV2 is a novel geminivirus that will likely be assigned to a new genus. Nevertheless, the molecular characterization of OpV2 shows that it shares several similarities with other geminiviruses including its genomic organization, the presence of conserved motifs at the origin of replication, intergenic region “iterons”, and characteristic Rep motifs. OpV2 has been identified in several species of *Opuntia*. Based on phylogenetic analysis, it is clear that OpV2 is very closely related to viruses in the genus *Curtovirus*. This is further supported by the presence of several recombination events in the Rep coding region between curtoviruses and OpV2 sequences.

It is plausible that the presence of Opuntia becurtovirus in cacti could be an example of spill-over from agricultural areas into native vegetation, given that spinach curly top Arizona virus was initially identified in spinach fields in Arizona, where it caused severe symptoms [93]. However, Opuntia becurtovirus was identified in three different states in the USA, suggesting it might have been circulating in the natural ecosystem for a longer time and that the spinach infection might have been a spillover or emergence event that originated in native uncultivated plants. More viral surveys with an ecological focus are needed to address this question. Nonetheless, our viral load analysis suggests that the viral load of Opuntia becurtovirus in cactus samples and experimentally inoculated *N. benthamiana* plants is low compared to those of OpV1 and OpV2.

Mixed infections are commonly reported for geminiviruses, and here we identified a few cactus samples infected with either OpV1 and OpV2 (*n* = 2) or Opuntia becurtovirus and either OpV2 (*n* = 2) or OpV1 (*n* = 6). Viral load quantification of the co-infected plants in comparison with cacti infected with only one of those viruses did not show any clear trend that would indicate that mixed infections favor any of the viruses. Further analysis of the OpV1 and OpV2 mixed infections in *N. benthamiana* plants showed that OpV2 is favored in mixed infections with OpV1, in that it displayed higher titers earlier in infections in the presence of OpV1 than it did in its absence.

Until very recently, cactus plants were mainly known to be infected by RNA viruses. Based on this study and our previous one [15], there are now three geminiviruses known to infect cacti. OpV1, OpV2 and Opuntia becurtovirus were found to infect cacti in the USA and OpV1 was found in cacti from Mexico. The fact that two novel geminiviruses and a distinct becurtovirus have been identified in an initial survey of cactus plants indicates that these plants may be hosts to other viruses. Further research efforts are needed to broaden our knowledge on viral diversity in cactus and the ways that these cactus viruses interact. The identification of Opuntia becurtovirus, which is most closely related to spinach curly top Arizona virus, may hint towards possible spill-overs between natural and agricultural areas and thus raises questions as to the extent to which viruses from cropping systems spill over into endemic uncultivated plants and vice-versa.

## Figures and Tables

**Table 1:** Summary of the Opuntia virus 2 and Opuntia becurtoviruses identified in this study with isolate names, accession numbers, genotypes, host species, collection dates, and locations. The instances where the cochineal insect was associated with any cactus host species are highlighted.

Virus	Host Species	ID	Accession Number	Genotype	Collection Year	Associated Scale Insect	Region of Collection
Opuntia becurtovirus	*Opuntia* spp.	ASU_PP13	MT840840	2	2018	SI_47	Arizona, USA
	*Opuntia* spp.	ASU_PP7	MT840839	2	2018		Arizona, USA
	*Opuntia martiniana*	DBG_38	MT840843	2	2018		Arizona, USA
	*Cylindropuntia echinocarpa*	DBG_80	MT840841	2	2018		Arizona, USA
	*Cylindropuntia spinosior*	DBG_86	MT840842	2	2018		Arizona, USA
	*Opuntia phaecantha*	LCM_23	MT840844	2	2006		Texas, USA
	*Opuntia stenopetala*	2014	MT840845	2	2015		Arizona, USA
	*Opuntia* spp.	S18_100	MT840861	2	2018		Arizona, USA
	*Opuntia* spp.	S18_101	MT840862	2	2018		Arizona, USA
	*Opuntia microdasys*	S18_12	MT840846	2	2018		Arizona, USA
	*Opuntia microdasys*	S18_14	MT840847	2	2018		Arizona, USA
	*Opuntia santa-rita*	S18_17	MT840870	2	2018		Arizona, USA
	*Opuntia basilaris*	S18_24	MT840848	2	2018		Arizona, USA
	*Opuntia engelmannii* var. *linguiformis*	S18_30	MT840849	2	2018		Arizona, USA
	*Opuntia* spp.	S18_34	MT840850	2	2018		Arizona, USA
	*Opuntia aciculata*	S18_40	MT840851	2	2018		Arizona, USA
	*Opuntia aciculata*	S18_54	MT840863	2	2018		Arizona, USA
	*Opuntia microdasys*	S18_56	MT840864	2	2018		Arizona, USA
	*Opuntia* spp.	S18_59_1	MT840852	2	2018	SI_68	Arizona, USA
		S18_59_2	MT840865	2	2018	SI_68	Arizona, USA
	*Opuntia engelmannii* var. *lindheimeri*	S18_69	MT840853	2	2018		Arizona, USA
	*Opuntia santa-rita*	S18_71_1	MT840854	2	2018		Arizona, USA
		S18_71_2	MT840855	2	2018		Arizona, USA
	*Opuntia phaeacantha*	S18_77	MT840856	2	2018		Arizona, USA
	*Opuntia microdasys*	S18_84_1	MT840857	2	2018		Arizona, USA
		S18_84_2	MT840858	2	2018		Arizona, USA
	*Opuntia engelmannii*	S18_89_1	MT840859	2	2018		Arizona, USA
		S18_89_2	MT840860	2	2018		Arizona, USA
	Scale insect	SI_47	MT840866	2	2018		Arizona, USA
	Scale insect	SI_68	MT840867	2	2018		Arizona, USA
	*Opuntia* sp.	TM3_2	MT840868	2	2018		Arizona, USA
	*Opuntia engelmannii*	UTH_RH6	MT840869	2	2018		Utah, USA
Opuntia virus 2	*Opuntia basilaris*	DBG_56	MT840871	8	2018		Arizona, USA
	*Opuntia basilaris*	DBG_57	MT840872	8	2018		Arizona, USA
	*Opuntia santa-rita*	DBG_61	MT840873	8	2018		Arizona, USA
	*Opuntia santa-rita*	DBG_62	MT840874	8	2018		Arizona, USA
	*Opuntia santa-rita*	DBG_63	MT840875	8	2018		Arizona, USA
	*Opuntia microdasys*	S18_12_1	MT840888	3	2018		Arizona, USA
		S18_12_2	MT840889	3	2018		Arizona, USA
	*Opuntia microdasys*	S18_14	MT840890	3	2018		Arizona, USA
	*Opuntia phaeacantha*	S18_25_1	MT840891	5	2018		Arizona, USA
		S18_25_2	MT840892	5	2018		Arizona, USA
	*Opuntia phaeacantha*	S18_26_1	MT840893	5	2018	SI_63	Arizona, USA
		S18_26_2	MT840894	5	2018	SI_63	Arizona, USA
		S18_26_3	MT840895	5	2018	SI_63	Arizona, USA
	*Opuntia phaeacantha*	S18_27	MT840896	3	2018	SI_64	Arizona, USA
	*Opuntia engelmannii*	S18_3_1	MT840876	6	2018		Arizona, USA
		S18_3_2	MT840877	6	2018		Arizona, USA
		S18_3_3	MT840878	6	2018		Arizona, USA
	*Opuntia engelmannii*	S18_4_1	MT840879	7	2018		Arizona, USA
		S18_4_2	MT840880	7	2018		Arizona, USA
		S18_4_3	MT840881	7	2018		Arizona, USA
	*Opuntia aciculata*	S18_40_1	MT840897	9	2018		Arizona, USA
		S18_40_2	MT840898	4	2018		Arizona, USA
		S18_40_3	MT840899	8	2018		Arizona, USA
	*Opuntia microdasys*	S18_41_1	MT840900	10	2018		Arizona, USA
		S18_41_2	MT840901	9	2018		Arizona, USA
	*Opuntia basilaris*	S18_5_1	MT840882	7	2018		Arizona, USA
		S18_5_2	MT840883	7	2018		Arizona, USA
	*Opuntia santa-rita*	S18_75_1	MT840902	1	2018		Arizona, USA
		S18_75_2	MT840903	1	2018		Arizona, USA
		S18_75_3	MT840904	1	2018		Arizona, USA
		S18_75_4	MT840905	11	2018		Arizona, USA
	*Opuntia santa-rita*	S18_8_1	MT840884	7	2018		Arizona, USA
		S18_8_2	MT840885	7	2018		Arizona, USA
		S18_8_3	MT840886	7	2018		Arizona, USA
		S18_8_4	MT840887	7	2018		Arizona, USA
	Scale insect	SI_63_1	MT840906	5	2018		Arizona, USA
		SI_63_2	MT840907	5	2018		Arizona, USA
	Scale insect	SI_64	MT840908	3	2018		Arizona, USA
	Scale insect	SI_70	MT840909	4	2018		Arizona, USA
	*Opuntia* sp.	TM_3_1	MT840910	2	2018		Arizona, USA
		TM_3_2	MT840911	2	2018		Arizona, USA
	*Opuntia santa-rita*	UTH_RH4	MT840912	8	2018		Utah, USA

**Table 2:** Summary of the 21 recombination events detected in the OpV2 sequences by RDP5 v5.5. The methods used to detect recombination were RDP (R), GENCONV (G), BOOTSCAN (B), MAXCHI (M), CHIMERA (C), SISCAN (S) and 3SEQ (T). The method with the highest *p*-value for each recombination event is bolded. Sites where the actual breakpoint is undetermined are marked with *. (T) denotes traces of recombination signals and (P) denotes partial evidence. Recombinant sequences marked with ^ indicate that the recombinant sequence may have been misidentified (one of the identified parents might be the recombinant). Please refer to the table for accession # of the genotypes.

Recombination EVENT	Region	Recombinant Sequence(s)	Minor Parental Sequence(s)	Major Parental Sequence(s)	Detection Methods	*p*-Value
1	2242–3220	Genotype 10	Genotype 9	Genotype 4	GBMCST	3.15 × 10^−42^
2~	2294–3203	Genotype 4	Genotype 3	Genotype 8	RGBMCST	1.91 × 10^−34^
3	2318–3210	Genotype 8	Genotype 8	Genotype 7	RGBMCT	1.12 × 10^−17^
			Genotype 7	Genotype 8		
4	531–2241	Genotype 3	Genotype 3	Genotype 5	RGBMCST	7.72 × 10^−22^
5	2325–3200	^Genotype 4	Genotype 3	Genotype 8	RBMCT	1.52 × 10^−15^
			Genotype 8	Genotype 3		
6	1051–2144	^Genotype 3	Unknown(Genotype 7)	Genotype 3	RBMCT	3.23 × 10^−8^
7	3014–3186	^Genotype 3	Genotype 5	Genotype 3	GMCST	7.31 × 10^−14^
		Genotype 4				
8	2147–173	^Genotype 6	Genotype 7	Genotype 6	RGBMCST	7.09 × 10^−18^
9	2585–2950	Genotype 6	Genotype 6	Genotype 3	RGBMCST	4.51 × 10^−13^
10	3009–3178	^Genotype 6	Genotype 1	Unknown (Genotype 7)	RGBMCS	9.77 × 10^−16^
11	1466–3037	Genotype 3	Genotype 5	Genotype 3	MCT	3.69 × 10^−6^
		Genotype 6[T]				
		Genotype 3[P]				
12	2611–3142	Genotype 11	Genotype 9	Genotype 1	RBMCST	7.37 × 10^−15^
13	3204–192	Genotype 3	Genotype 1	Genotype 5	RBT	1.93 × 10^−3^
14	1740–2322	^Genotype 7	Unknown (Genotype 5)	Genotype 7	RGMCST	1.78 × 10^−11^
15	2493–3161	^Genotype 9	Genotype 8	Unknown (Genotype 7)	GBMCS	2.69 × 10^−18^
		Genotype 10				
16	3169 *–1027	^Genotype 3	Genotype 3	Genotype 6	RGBMCST	2.95 × 10^−9^
17	2008–2991	^Genotype 1	Genotype 5	Genotype 8	RGBMCS	5.19 × 10^−15^
18	1034–1650 *	^Genotype 8	Genotype 6	Genotype 6	RGMCS	9.12 × 10^−11^
19	1862–2995 *	^Genotype 6	Genotype 5	Genotype 8	RGMCST	2.68 × 10^−12^
20	1907–2049	^Genotype 3	Genotype 4	Genotype 4	RGMCS	1.11 × 10^−6^
				Genotype 10		
21	349–606	^Genotype 3	Genotype 2	Genotype 3	RBT	5.23 × 10^−4^

**Table 3:** Summary of the three recombination events detected in the Opuntia becurtovirus by RDP5 v.5.5. The methods used to detect recombination were RDP (R) GENCONV (G), BOOTSCAN (B), MAXCHI (M), CHIMERA (C), SISCAN (S), and 3SEQ (T). The method with the highest *p*-value for each recombination event is bolded. Sites where the actual breakpoint is undetermined are marked with *. Recombinant sequences marked with ^ indicate that the recombinant sequence may have been misidentified (one of the identified parents might be the recombinant).

Recombination Event	Region	Recombinant Sequence(s)	Minor Parental Sequence(s)	Major Parental Sequence(s)	Detection Methods	*p*-Value
1	2790–1165	^MT840851	MT840856	Unknown	RBMCST	1.29 × 10^−7^
2	1546–2590 *	^MT840860	MT840870	MT840848	MCST	2.86 × 10^−11^
		MT840859	MT840856			
3~	1525–2036	^MT840839	MT840841	MT840868	RBCS	4.86 × 10^−7^
		MT840866				

**Table 4:** Summary of the seven recombination events from a dataset of representative sequences of OpV2 and Opuntia becurtovirus, with representatives from the genera *Becurtoviru*s and *Curtovirus*, as detected by RDP5 5.5 [63]. The methods used to detect recombination were RDP (R) GENCONV (G), BOOTSCAN (B), MAXCHI (M), CHIMERA (C), SISCAN (S), and 3SEQ (T). The method with the highest *p*-value for each recombination event is bolded. Sites where the actual breakpoint is undetermined are marked with *. Recombinant sequences marked with ^ indicate that the recombinant sequence may have been misidentified (one of the identified parents might be the recombinant).

Recombination Event	Region	Recombinant Sequence(s)	Minor Parental Sequence(s)	Major Parental Sequence(s)	Detection Methods	*p*-Value
1	726–1474	^KX529650 curtovirus	Unknown (AF379637 curtovirus)	AY134867 curtovirus	RGBMCST	8.79 × 10^−33^
		EU921828 curtovirus		JN817383 curtovirus		
2~	2719–3418 *	^AF379637 curtovirus	MT840871 OpV2	AY134867 curtovirus	RGBMCS	2.56 × 10^−24^
				JN817383 curtovirus		
				U02311 curtovirus		
3	729 *–1498 *	^GU734126 curtovirus	HQ443515 becurtovirus	U49907 curtovirus	RMCST	1.22 × 10^−14^
4	2978–3374	^U02311 curtovirus	MT840871 OpV2	JN817383 curtovirus	RGBMCST	5.13 × 10^−46^
5	2843 *–3375	^U49907 curtovirus	Unknown (GU734126 curtovirus)	MT840871 OpV2	RMCT	1.08 × 10^−11^
			Unknown (MT840871 OpV2)	GU734126 curtovirus		
6	727 *–928	EU921828 curtovirus	JN817383 curtovirus	KX529650 curtovirus	GBMCST	4.57 × 10^−12^
			AY134867 curtovirus			
7	2073–2502	^JN817383 curtovirus	U02311 curtovirus	AY134867 Becurtovirus	RGBMST	4.11 × 10^−12^

## Data Availability

Sequence determined as part of this study have been deposited in GenBank under accession #s MT840839—MT840912.

## Supplementary Materials

The following are available online at https://www.mdpi.com/article/10.3390/v13040694/s1, Supplementary Table S1: Primer pairs used for real-time quantitative PCR analysis of the OpV1, OpV2 and Opuntia becurtovirus, including the sequence and melting temperature. Supplementary Table S2: Summary of the rolling circle, SF3, and Walker motifs identified in the Rep amino acid sequences encoded by the Opuntia Virus 2, Opuntia becurtovius, and spinach curly top Arizona virus sequences. Supplementary Figure S1: (A) Southern blot showing replicative forms of OpV2 infectious clone from *N. benthamiana* plants inoculated with OpV2 and Opuntia becurtovirus, including a non-inoculated control and positive control with an amplicon of linearized full-length OpV2 genome (5 ng). The gel images on the top show the DNA (5 µg) loaded for each sample. CCC—circular covalently closed; SC—super coiled dsDNA; SS—single-stranded. (B) Transmission electron micrographs (negatively stained with 2% uranyl acetate) of geminate particles (shown with white arrows) from OpV2-inoculated plants. Bottom image scale bar is 100 nm and the top zoomed section scale bar is 50 nm. Supplementary Figure S2: Bar graph represents the viral load quantification of two *N. benthamiana* plants infected with Opuntia becurtovirus at 45 days post-inoculation, acquired by real-time quantitative PCR. Standard deviation represents triplicate reactions. Supplementary Data S1: Pairwise sequence identity of the full-length genome sequences and encoded amino acid sequences of the Rep and CP from the 42 full-length OpV2 genomes. Supplementary Data S2: Pairwise sequence identity of the full-length genome sequences and encoded amino acid sequences of the Rep and CP of OpV2 genotypes and Opuntia becurtovirus, together with representatives of the geminiviruses genera and unclassified geminiviruses. Supplementary Data S3: Pairwise sequence identity of the full-length genome sequences and encoded amino acid sequences of the Rep and CP from the 32 cactus-derived becurtovirus genomes.
