# Supplementary material for: New World Cactaceae Plants Harbor Diverse Geminiviruses
Source: Viruses. 2021 Apr 16;13(4):694. doi: 10.3390/v13040694 (PMC8073023; doi:10.3390/v13040694)
Supplement: Supplementary file 1 [file viruses-13-00694-s001.zip › viruses-1163701-supplementary/Supplementary_Table_1.pdf]

Primers	Target	Sequence	Annealing Temp.
OpV1_q_CP_F<br>OpV1_q_CP_R	Opuntia virus 1	5’-CACTTATGTTAGTATTAGAGGGGAGCTG-3’<br>5’-CTATATCAAACGTATCTCCTCTGACTG-3’	60
OpV2_q_Rep_F<br>OpV2_q_Rep_R	Opuntia virus 2	5’-GGATTTGATGGTAGTGAAAGATTGG-3’<br>5’-CTCCAAAAAGATTCAGAGTTTCAGC-3’	58
Bec_q_Rep_F<br>Bec_q_Rep_R	Opuntia becurtovirus	5’-GTAGTGATGTCTTCCTAATGATCTGG-3’<br>5’-CTAATGATCTGTCTTCTACCAGGAAC-3’	56
