# Supplementary material for: New World Cactaceae Plants Harbor Diverse Geminiviruses
Source: Viruses. 2021 Apr 16;13(4):694. doi: 10.3390/v13040694 (PMC8073023; doi:10.3390/v13040694)
Supplement: Supplementary file 1 [file viruses-13-00694-s001.zip › viruses-1163701-supplementary/Supplementary_Table_2.pdf]

Geminivirus	Accession #	Motif I	Motif II	GRS	Motif III	Walker A	Walker B	Motif C
Spinach curly top Arizona virus	HQ443515	FLTYS	HTHCIF	LFFNIILPDGRTIHPRIDGLNAP	YITKE	GRSPNRLGDSNL	VIDDI	IGCN
Opuntia becurtovirus ASU PP7	MT840839	FLTYS	HTHCIF	LFFNIILPDGRTIHPRIDGLNAP	YITKE	GRSPNRLGNSNM	VIDDI	IGCN
Opuntia becurtovirus ASU PP13	MT840840	FLTYS	HTHCIF	LFFNIILPDGRIIHPRVDGLNAP	YITKE	GRSPNRLGNSNM	VIDDI	IGCN
Opuntia becurtovirus DBG 80	MT840841	FLTYS	HTHCIF	LFFNIILPDGRIIHPRVDGLNAP	YITKE	GRSPNRIGNSNM	VIDDI	IGCN
Opuntia becurtovirus DBG 86	MT840842	FLTYS	HTHCIF	LFFNIILPDGRIIHPRVDGLNAP	YITKE	GRSPNRIGNSNM	VIDDI	IGCN
Opuntia becurtovirus DBG 38	MT840843	FLTYS	HTHCIF	LFFNIILPDGRIIHPRVDGLNAP	YITKE	GRSPNRIGNSNL	VIDDI	IGCN
Opuntia becurtovirus LCM 23	MT840844	FLTYS	HTHCIF	LFFNIILPDGRIIHPRVDGLNAP	YITKE	GRSPNRLGNSNM	VIDDI	IGCN
Opuntia becurtovirus Martin 2014	MT840845	FLTYS	HTHCIF	LFFNIILPDGRIIHPRVDGLNAP	YITKE	GRSPNRLGNSNM	VIDDI	IGCN
Opuntia becurtovirus S18 12	MT840846	FLTYS	HTHCIF	LFFNIILPDGRIIHPRVDGLNAP	YITKE	GRSPNRLGNSNM	VIDDI	IGCN
Opuntia becurtovirus S18 14	MT840847	FLTYS	HTHCIF	LFFNIILPDGRVIHPRVDGLNAP	YITKE	GRSPNRIGNSNL	VIDDI	IGCN
Opuntia becurtovirus S18 24	MT840848	FLTYS	HTHCIF	LFFNIILPDGRIIHPRVDGLNAP	YITKE	GRSPNRLGNSNM	VIDDI	IGCN
Opuntia becurtovirus S18 30	MT840849	FLTYS	HTHCIF	LFFNIILPDGRIIHPRVDGLNAP	YITKE	GRSPNRIGNSNL	VIDDI	IGCN
Opuntia becurtovirus S18 34	MT840850	FLTYS	HTHCIF	LFFNIILPDGRIIHPRVDGLNAP	YITKE	GRSPNRLGNSNM	VIDDI	IGCN
Opuntia becurtovirus S18 40	MT840851	FLTYS	HTHYIF	LFFNIILPDGRVIHPRVDGLNAP	YITKE	GRSPNRIGNSNL	VIDDI	IGCN
Opuntia becurtovirus S18 59 1	MT840852	FLTYS	HTHCIF	LFFNIILPDGRIIHPRVDGLNAP	YITKE	GRSPNRLGNSNM	IIDDI	IGCN
Opuntia becurtovirus S18 69	MT840853	FLTYS	HTHCIF	LFFNIILPDGRIIHPRVDGLNAP	YITKE	GRSPNRIGNSNM	VIDDI	IGCN
Opuntia becurtovirus S18 71 1	MT840854	FLTYS	HTHCIF	LFFNIILPDGRIIHPRVDGLNAP	YITKE	GRSPNRIGNSNM	VIDDI	IGCN
Opuntia becurtovirus S18 71 2	MT840855	FLTYS	HTHCIF	LFFNIILPDGRIIHPRVDGLNAP	YITKE	GRSPNRIGNSNM	VIDDI	IGCN
Opuntia becurtovirus S18 77	MT840856	FLTYS	HTHCIF	LFFNIILPDGRTIHPRVDGLNAP	YITKE	GRSPNRIGNSNL	VIDDI	IGCN
Opuntia becurtovirus S18 84 1	MT840857	FLTYS	HTHCIF	LFFNIILPDGRIIHPRVDGLNAP	YITKE	GRSPNRLGNSNM	VIDDI	IGCN
Opuntia becurtovirus S18 84 2	MT840858	FLTYS	HTHCIF	LFFNIILPDGRIIHPRVDGLNAP	YITKE	GRSPNRLGNSNM	VIDDI	IGCN
Opuntia becurtovirus S18 89 1	MT840859	FLTYS	HTHCIF	LFFNIILPDGRVIHPRVDGLNAP	YITKE	GRSPNRIGNSNL	VIDDI	IGCN
Opuntia becurtovirus S18 89 2	MT840860	FLTYS	HTHCIF	LFFNIILPDGRVIHPRVDGLNAP	YITKE	GRSPNRIGNSNL	VIDDI	IGCN
Opuntia becurtovirus S18 100	MT840861	FLTYS	HTHCIF	LFFNIILPDGRIIHPRVDGLNAP	YITKE	GRSPNRIGNSNM	VIDDI	IGCN
Opuntia becurtovirus S18 101	MT840862	FLTYS	HTHCIF	LFFNIILPDGRIIHPRVDGLNAP	YITKE	GRSPNRIGNSNM	VIDDI	IGCN
Opuntia becurtovirus S18 54	MT840863	FLTYS	HTHCIF	LFFNIILPDGRIIHPRVDGLNAP	YITKE	GRSPNRIGNSNL	VIDDI	IGCN
Opuntia becurtovirus S18 56	MT840864	FLTYS	HTHCIF	LFFNIILPDGRIIHPRVDGLNAP	YITKE	GRSPNRLGNSNL	VIDDI	IGCN
Opuntia becurtovirus S18 59 2	MT840865	FLTYS	HTHCIF	LFFNIILPDGRIIHPRVDGLNAP	YITKE	GRSPNRLGNSNM	IIDDI	IGCN
Opuntia becurtovirus SI 47	MT840866	FLTYS	HTHCIF	LFFNIILPDGRIIHPRVDGLNAP	YITKE	GRSPNRLGNSNM	VIDDI	IGCN
Opuntia becurtovirus SI 68	MT840867	FLTYS	HTHCIF	LFFNIILPDGRIIHPRVDGLNAP	YITKE	GRSPNRLGNSNM	VIDDI	IGCN
Opuntia becurtovirus TM3 2	MT840868	FLTYS	HTHCIF	LFFNIILPDGRVIHPRVDGLNAP	YITKE	GRSPNRLGNSNM	VIDDI	IGCN
Opuntia becurtovirus UTH RH6	MT840869	FLTYS	HTHCIF	LFFNIILPDGRIIHPRVDGLNAP	YITKE	GRSPNRIGNSNL	VIDDI	IGCN
Opuntia becurtovirus S18 17	MT840870	FLTYS	HTHCIF	LFFNIILPDGRIIHPRVDGLNAP	YITKE	GRSPNRIGNSNL	VIDDI	IGCN
Opuntia virus 2 DBG 56	MT840871	FLTYP	HLHILL	RLFDLVSPTRSAHFHPNIQGAKS	YIDKD	GRSARGGQQTAN	VIDDV	WQSN
Opuntia virus 2 DBG 57	MT840872	FLTYP	HLHILL	RLFDLVSPTRSAHFHPNIQGAKS	YIDKD	GRSARGGQQTAN	VIDDV	WQSN
Opuntia virus 2 DBG 61	MT840873	FLTYP	HLHILL	RLFDLVSPTRSAHFHPNIQGAKS	YIDKD	GRSARGGQQTAN	VIDDV	WQSN
Opuntia virus 2 DBG 62	MT840874	FLTYP	HLHILL	RLFDLVSPTRSAHFHPNIQGAKS	YIDKD	GRSARGGQQTAN	VIDDV	WQSN
Opuntia virus 2 DBG 63	MT840875	FLTYP	HLHILL	RLFDLVSPTRSAHFHPNIQGAKS	YIDKD	GRSARGGQQTAN	VIDDV	WQSN
Opuntia virus 2 S18 3 1	MT840876	FLTYP	HLHVLI	RLFDLVSPTRSAHFHPNIQGAKS	YIDKD	GRSARGGCHKAN	VIDDV	WQSN
Opuntia virus 2 S18 3 2	MT840877	FLTYP	HLHVLI	RLFDLVSPTRSAHFHPNIQGAKS	YIDKD	GRSARGGCHKAN	VIDDV	WQSN
Opuntia virus 2 S18 3 3	MT840878	FLTYP	HLHVLI	RLFDLVSPTRSAHFHPNIQGAKS	YIDKD	GRSARGGCHKAN	VIDDV	WQSN
Opuntia virus 2 S18 4 1	MT840879	FLTYP	HLHILI	RLFDLVSPTRSAHFHPNIQGAKS	YIDKD	GRSARGGSQKAN	VIDDV	WQSN
Opuntia virus 2 S18 4 2	MT840880	FLTYP	HLHILI	RLFDLVSPTRSAHFHPNIQGAKS	YIDKD	GRSARGGSQKAN	VIDDV	WQSN
Opuntia virus 2 S18 4 3	MT840881	FLTYP	HLHILI	RLFDLVSPTRSAHFHPNIQGAKS	YIDKD	GRSARGGSQKAN	VIDDV	WQSN
Opuntia virus 2 S18 5 1	MT840882	FLTYP	HLHILI	RLFDLVSPTRSAHFHPNIQGAKS	YIDKD	GRSARGGSQKAN	VIDDV	WQSN
Opuntia virus 2 S18 5 2	MT840883	FLTYP	HLHILI	RLFDLVSPTRSAHFHPNIQGAKS	YIDKD	GRSARGGSQKAN	VIDDV	WQSN
Opuntia virus 2 S18 8 1	MT840884	FLTYP	HLHILI	RLFDLVSPTRSAHFHPNIQGAKS	YIDKD	GRSARGGSQKAN	VIDDV	WQSN
Opuntia virus 2 S18 8 2	MT840885	FLTYP	HLHILI	RLFDLVSPTRSAHFHPNIQGAKS	YIDKD	GRSARGGSQKAN	VIDDV	WQSN
Opuntia virus 2 S18 8 3	MT840886	FLTYP	HLHILI	RLFDLVSPTRSAHFHPNIQGAKS	YIDKD	GRSARGGSQKAN	VIDDV	WQSN
Opuntia virus 2 S18 8 4	MT840887	FLTYP	HLHILI	RLFDLVSPTRSAHFHPNIQGAKS	YINKD	GRSARGGSQKAN	VIDDV	WQSN
Opuntia virus 2 S18 12 1	MT840888	FLTYP	HLHVLI	RLFDLVSPTRSAHFHPNIQGAKS	YIDKD	GRSARGGCQKAN	VIDDV	WQSN
Opuntia virus 2 S18 12 2	MT840889	FLTYP	HLHVLI	RLFDLVSPTRSAHFHPNIQGAKS	YIDKD	GRSARGGCQKAN	VIDDV	WQSN
Opuntia virus 2 S18 14	MT840890	FLTYP	HIHVLI	RLFDLVSPTRSAHFHPNIQGAKS	YIDKD	GRSARGGSQKAN	VIDDV	WQSN
Opuntia virus 2 S18 25 1	MT840891	FLTYP	HLHVLI	RLFDLVSPTRSAHFHPNIQGAKS	YIDKD	GRSARGGCQKAN	VIDDV	WQSN
Opuntia virus 2 S18 25 2	MT840892	FLTYP	HLHVLI	RLFDLVSPTRSAHFHPNIQGAKS	YIDKD	GRSARGGCQKAN	VIDDV	WQSN
Opuntia virus 2 S18 26 1	MT840893	FLTYP	HLHVLI	RLFDLVSPTRSAHFHPNIQGAKS	YIDKD	GRSARGGCQKAN	VIDDI	WQSN
Opuntia virus 2 S18 26 2	MT840894	FLTYP	HLHVLI	RLFDLVSPTRSAHFHPNIQGAKS	YIDKD	GRSARGGCQKAN	VIDDV	WQSN
Opuntia virus 2 S18 26 3	MT840895	FLTYP	HLHVLI	RLFDLVSPTRSAHFHPNIQGAKS	YIDKD	GRSARGGCQKAN	VIDDI	WQSN
Opuntia virus 2 S18 27	MT840896	FLTYP	HLHVLI	RLFDLVSPTRSAHFHPNIQGAKS	YIDKD	GRSARGGCPKAN	VIDDV	WQSN
Opuntia virus 2 S18 40 1	MT840897	FLTYP	HLHILL	RLFDLVSPTRSAHFHPNIQGAKS	YIDKD	GRSARGGQQTAN	VIDDV	WQSN
Opuntia virus 2 S18 40 2	MT840898	FLTYP	HIHVLI	RLFDLVSPTRSAHFHPNIQGAKS	YIDKD	GRSARGGSQKAN	VIDDV	WQSN
Opuntia virus 2 S18 40 3	MT840899	FLTYP	HLHILL	RLFDLVSPTRSAHFHPNIQGAKS	YIDKD	GRSARGGQQTAN	VIDDV	WQSN
Opuntia virus 2 S18 41 1	MT840900	FLTYP	HLHILL	RLFDLVSPTRSAHFHPNIQGAKS	YIDKD	GRSARGGQQTAN	VIDDV	WQSN
Opuntia virus 2 S18 41 2	MT840901	FLTYP	HLHILL	RLFDLVSPTRSAHFHPNIQGAKS	YIDKD	GRSARGGQQTAN	VIDDV	WQSN
Opuntia virus 2 S18 75 1	MT840902	FITYP	HLHVLI	RLFDLVSPTRSTHFHPNIQGAKS	YIDKD	GRSARGGCQKAN	VIDDV	WQSN
Opuntia virus 2 S18 75 2	MT840903	FITYP	HLHVLI	RLFDLVSPTRSTHFHPNIQGAKS	YIDKD	GRSARGGCQKAN	VIDDV	WQSN
Opuntia virus 2 S18 75 3	MT840904	FITYP	HLHVLI	RLFDLVSPTRSAHFHPNIQGAKS	YIDKD	GRSARGGCQKAN	VIDDV	WQSN
Opuntia virus 2 S18 75 4	MT840905	FLTYP	HLHVLL	RLFDLVSPTRSAHFHPNIQGAKS	YIDKD	GRSARGGQQTAN	VIDDV	WQSN
Opuntia virus 2 SI 63 1	MT840906	FLTYP	HLHVLI	RLFDLVSPTRSAHFHPNIQGAKS	YIDKD	GRSARGGCKKAN	VIDDV	WQSN
Opuntia virus 2 SI 63 2	MT840907	FLTYP	HLHVLI	RLFDLVSPTRSAHFHPNIQGAKS	YIDKD	GRSARGGCQKAN	VIDDV	WQSN
Opuntia virus 2 SI 64	MT840908	FLTYP	HLHVLI	RLFDLVSPTRSAHFHPNIQGAKS	YIDKD	GRSARGGCPKAN	VIDDV	WQSN
Opuntia virus 2 SI 70	MT840909	FLTYP	HIHVLI	RLFDLVSPTRSAHFHPNIQGAKS	YIDKD	GRSARGGSQKAN	VIDDV	WQSN
Opuntia virus 2 TM cacti 3 1	MT840910	FITYP	HLHVLI	RLFDLVSPTRSAHFHPNIQGAKS	YIDKD	GRSARGGCQKAN	VIDDV	WQSN
Opuntia virus 2 TM cacti 3 2	MT840911	FITYP	HLHVLI	RLFDLVSPTRSAHFHPNIQGAKS	YIDKD	GRSARGGCQKAN	VIDDV	WQSN
Opuntia virus 2 UTH RH4	MT840912	FLTYP	HLHILL	RLFDLVSPTRSAHFHPNIQGAKS	YIDKD	GRSARGGQQTAN	VIDDV	WQSN
