# Supplementary figures and images for: New World Cactaceae Plants Harbor Diverse Geminiviruses
Source: Viruses. 2021 Apr 16;13(4):694. doi: 10.3390/v13040694 (PMC8073023; doi:10.3390/v13040694)

A

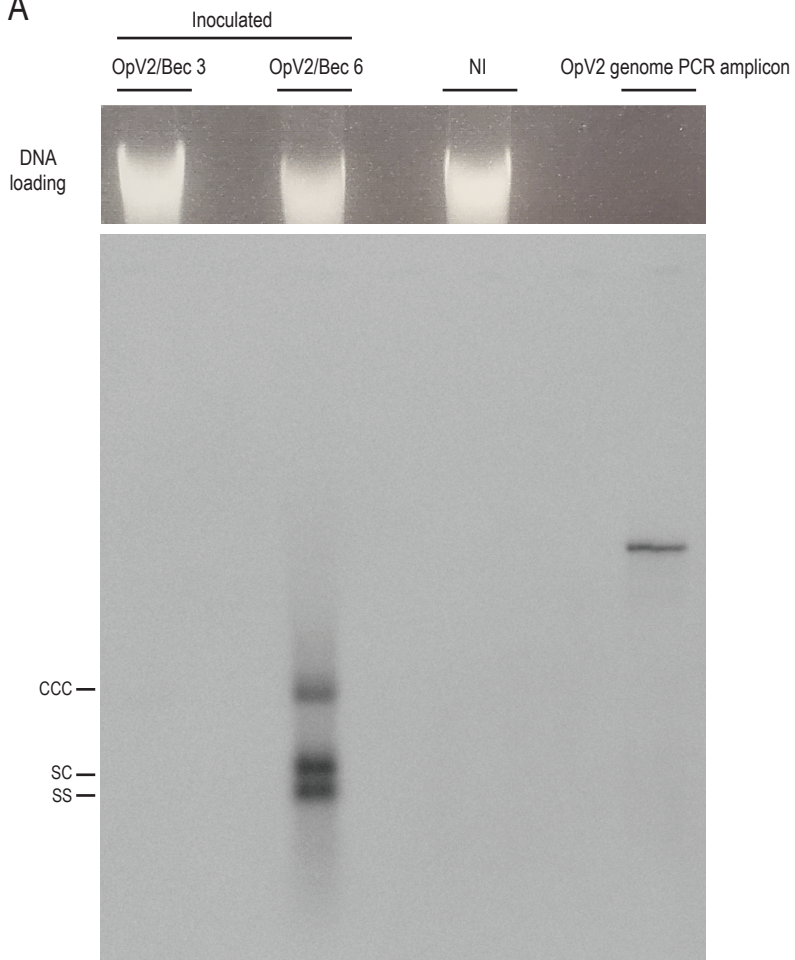

B

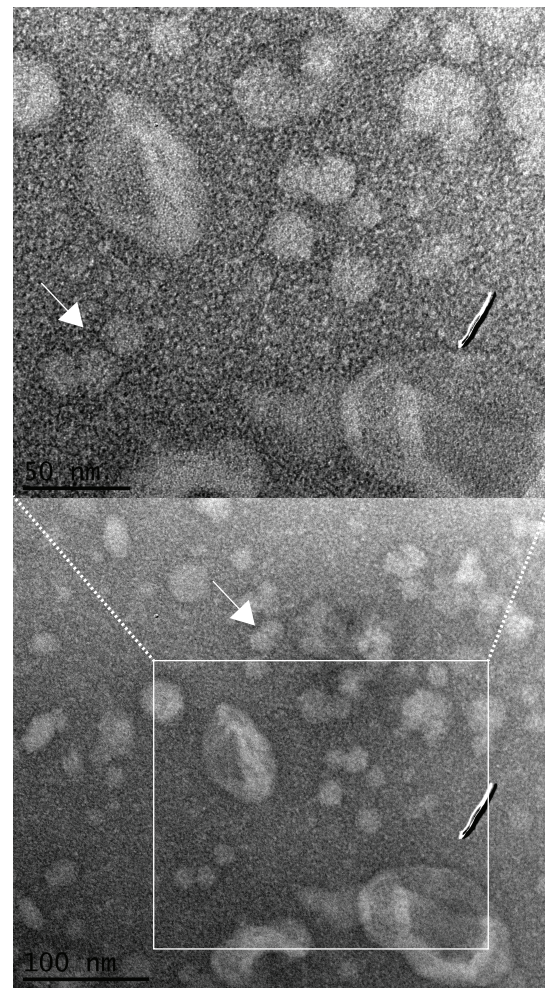

Supplement: Supplementary file 1 [file viruses-13-00694-s001.zip › viruses-1163701-supplementary/Supplementary_Figure_1.pdf]

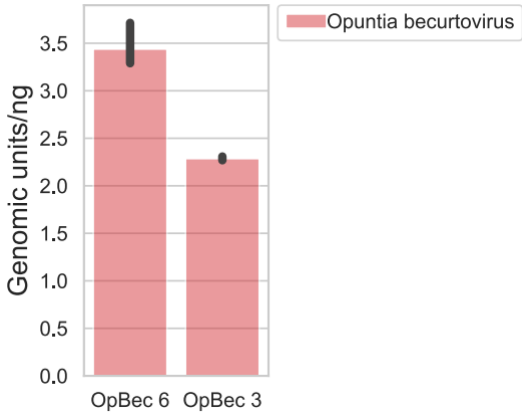

Supplement: Supplementary file 1 [file viruses-13-00694-s001.zip › viruses-1163701-supplementary/Supplementary_Figure_2.pdf]
